# A two-wave survey study examining the impact of different sources of pregnancy information on pregnancy-related anxiety among Swedish women

**DOI:** 10.18332/ejm/197169

**Published:** 2025-01-17

**Authors:** Femke Geusens, Alkistis Skalkidou

**Affiliations:** 1 Department of Women’s and Children’s Health, Uppsala University, Uppsala, Sweden; 2 REALIFE Research Group, Research Unit Woman and Child, Department of Development and Regeneration, Leuven, Belgium

**Keywords:** pregnancy-related anxiety, online health information seeking, maternal mental health, information seeking behavior, maternal behavior

## Abstract

**INTRODUCTION:**

During pregnancy, women rely on a variety of sources to obtain information. However, not all of these sources are equally reliable, and there is the concern that especially online information-seeking may increase pregnancy-related anxiety. This study examines to what extent different sources of pregnancy information are associated with concurrent pregnancy-related anxiety (RQ1) and changes in pregnancy-related anxiety throughout the pregnancy (RQ2).

**METHODS:**

This study was integrated into the ongoing Swedish Mom2B study (sub-study data collection: December 2022–April 2024), where women complete weekly questionnaires via a research app. Each trimester, they received questions about their use of information sources and pregnancy-related anxiety.

**RESULTS:**

Our sample consisted of 751 pregnant women (273 with at least two waves of data). Using the midwife (β= -0.14, p<0.001; 95% CI: -3.32 – -1.13) or social circle (β= -0.08, p<0.05; 95% CI: -2.83 – -0.07) as a source of pregnancy-and childbirth-related information was associated with lower levels of pregnancy-related anxiety. In contrast, reliance on online sources for information was associated with higher levels of anxiety (β=0.14, p<0.001; 95% CI: 1.52–5.03). Except for (e-)books, which lowered the odds of improving anxiety (OR=0.62, p<0.01; 95% CI: 0.45–0.85), none of the information sources predicted changes in pregnancy-related anxiety over time.

**CONCLUSIONS:**

Not all information sources play an equal role in relation to pregnancy-related anxiety. Interpersonal sources in particular may help mitigate anxiety. However, future research with more nuanced methodologies and shorter measurement intervals could clarify possible causal relationships and refine our understanding of how various information sources affect pregnancy-related anxiety over time.

## INTRODUCTION

During pregnancy, women’s information needs increase^1^. Knowledge and information are crucial for ensuring their own and their developing babies’ health and well-being. Prior research has indicated that women use a variety of information sources, including pregnancy care providers, individuals within their social circle, the internet, apps, and other media sources^2-4^. Recent studies even indicate that women are equally likely to rely on the Internet for pregnancy information, as on their pregnancy care provider^3^. However, not all of these sources are equally reliable. Research on the quality of different information sources has repeatedly shown that online sources are not of high quality^1,5,6^. A recent review indicated that many women acknowledge that the internet is not a reliable source of information, yet they do use this as an information source^4^. In addition, online sources, especially forums and social media, also provide social and emotional support^4^. Hence, they are used not only to meet informational needs but also reassurance needs.

Many pregnancy care providers note that their patients are using the internet as an information source, and worry about its effects on their mental health, specifically anxiety. However, this relationship is not well studied. One qualitative interview study found that women sometimes find it hard to stop searching the web for information, which can increase feelings of anxiety^7^. Similarly, two recent survey studies found that increased engagement with ‘mommy influencers’ on Instagram was associated with greater anxiety in mothers of young children^8^ and pregnancy-related anxiety (PRA) predicted cyberchondria, defined as the excessive online searching for health information resulting in a negative emotional reaction^9^. In contrast, one study found that online information searching reduced anxiety among women hospitalized for pregnancy complications^10^. The authors suggested that this may partially be explained by the fact that they found information that was in line with information received from care providers, and partially because the internet helps to connect to a community that can provide support^10^. Yet another study found that both relying on media sources and interpersonal sources increased the anxiety of pregnant women undergoing invasive prenatal testing, probably because of the untrustworthiness of these sources^11^. It is noteworthy that only relying on healthcare providers was associated with lower anxiety levels^11^.

These findings raise the question to what extent different information sources are actually helpful to women in reducing anxiety. This study aims thus to examine to what extent different sources of pregnancy information are associated with concurrent levels of PRA at any time during the pregnancy (RQ1), as well as changes in PRA throughout the pregnancy (RQ2). Overall, we expect that a greater reliance on healthcare providers as an information source will be associated with lower levels of PRA and decreasing PRA over time, and greater reliance on online sources will be associated with higher levels of PRA and increasing PRA over time. It is unclear what to expect regarding the associations between interpersonal sources or parenting and pregnancy books as information sources and PRA.

## METHODS

### Study design and setting

Participants were drawn from the Swedish Mom2B study (For the study’s protocol see: citation 12). Mom2B combines automatically collected digital phenotyping data (e.g. internet and data use, GPS movement data, Facebook activity), weekly self-report questionnaires that participants can answer directly in the app, and objective information from national health registers (e.g. level of education, employment, income status, healthcare diagnoses). The larger project was launched in November 2019; the present sub-study was added on 25 December 2022. Data collection is still ongoing and we are presenting data collected between December 2022 and April 2024 in this work. The main goal of Mom2B is to assess the accuracy of advanced machine learning and deep learning methods in predicting the development of peripartum depression. To date, the data have been used to explore user experiences of the app, predict antenatal depression onset using machine learning algorithms^12-14^, and assess depressive and anxiety symptoms, as well as well-being and life changes in pregnant women during the COVID-19 pandemic in Sweden^15^.

### Participants

Swedish-speaking women aged ≥18 years who owned a smartphone and were either pregnant or within three months postpartum were eligible to participate in the study by registering and providing consent in the Mom2B app. Women were recruited through convenience sampling via social media advertising, as well as brochures and posters in midwifery clinics across Sweden. The goal for the full study is to recruit at least 5000 participants^12^. Power analysis indicated that we needed at least 244 participants for this sub-study [G*Power 3.1.9.7; A priori power analysis, linear multiple regression: R^2^ deviation from zero, small effect size |f²|=0.05, α err prob=0.05, Power (1-β err prob)=0.80, four predictors]. The same data were used to address separate research questions: a larger sample was used for the cross-sectional design (RQ1), while a subset of this larger sample was utilized for the longitudinal design (RQ2). This subset allowed us to track participants over time to explore changes and patterns, while the full sample provided a broader snapshot for cross-sectional analysis.

### Data sources and measurement

Study participants installed a smartphone application where they can track their pregnancy symptoms and their mood, as well as donate data through surveys and other methods, such as audio recordings and GPS tracking^12^. To authenticate responses, women’s pregnancy was checked against the Swedish pregnancy register upon registration. For this sub-study, we added questions on PRA and sources of pregnancy and childbirth information to the running rotation of Mom2B surveys. Each week, women were presented with a few short questionnaires on a variety of topics, related to the main study and sub-studies in the project. The questions for this sub-study were presented three times: once per pregnancy trimester, and we combined cross-sectional data and longitudinal data from two consecutive pregnancy trimesters for the analyses.

Because women can enter the study at any time during their pregnancy or first three months postpartum, not all women will have been presented with all questionnaires. For the longitudinal dataset, we included data from the first two consecutively completed pregnancy trimesters. This means that if women had completed surveys in all three trimesters (n=54; 19.8%), we only used their data from the first and second trimesters, not their third pregnancy trimester. This resulted in 99 (36.3%) women whose baseline data are from their first pregnancy trimester, and follow-up data from their second trimester, and 174 (63.7%) women whose baseline data are from their second pregnancy trimester, and follow-up data from their third trimester. This results in a total sample of 273 women who had provided data on the main measurements in two consecutive pregnancy trimesters, and who were included in the longitudinal dataset. The other 487 women in the sample entered the study in their third trimester or only provided data in one pregnancy trimester, or in non-consecutive trimesters (i.e. in their first and third trimester, but not their second trimester).

### Variables

This study addresses pregnancy-related anxiety as the outcome variable, sources of pregnancy and childbirth information as the exposure variables, and sociodemographic variables as potential confounders.

### Pregnancy-related anxiety

This was measured using the Brunton et al.^16,17^ validated Pregnancy-Related Anxiety Scale (PrAS), which we translated into Swedish. PrAS consists of 32 items with response options ranging from 1 (not at all) to 4 (very often): 6 items on childbirth concerns, 5 on body image, 3 on attitudes towards childbirth (reverse-scored), 7 on worry about the self, 4 on acceptance of pregnancy (reverse scored), 4 on attitudes towards medical staff, 3 on avoidance of vaginal birth, and 3 on concerns about the baby’s health and wellbeing. A sum score of all items was calculated (Cronbach’s α=0.92), with a hypothetical range of 32–128. A score of ≥75.5 indicates clinical levels of anxiety^16^. PRA was measured once per pregnancy trimester.

### Sources of pregnancy and childbirth information

These were measured with a self-developed scale. We asked individuals: ‘When you are looking for information about pregnancy or childbirth, where are you likely to you seek your information?’ with response options for each source ranging from 1 (extremely unlikely) to 5 (extremely likely). We then provided them with 19 potential sources of information, based on the literature and discussions between the first author (a communication scientist) and pregnant women in her social circle: healthcare providers (i.e. your midwife, your obstetrician/gynecologist, your general practitioner), social circle (i.e. your mother or grandmother, your partner, your siblings or cousins, your best friend, other moms in your environment; α = 0.64), online sources (i.e. informational websites, forum websites, blogs, smartphone apps, Instagram, Facebook, Reddit, TikTok, YouTube; α=0.77) and literature (i.e. physical books, e-books; Spearman-Brown coefficient=0.62). We included several healthcare providers in the questionnaire because we aim to conduct cross-cultural comparisons using this dataset in future studies. However, because of the midwifery-led-care model of Sweden, only the midwife was included as a healthcare provider in the analyses in this work. The scale was not pilot-tested, but face validity was established through discussion in the research group (consisting of nurses, midwives, obstetricians, psychiatrists, psychologists, and a communication scientist). For each of the information sources (except the midwife as the sole healthcare provider), a mean score was calculated. Sources of pregnancy and childbirth information were measured once per pregnancy trimester.

### Sociodemographic variables

These were assessed when registering for the study. Age was ascertained with an open question. The pregnancy trimester was measured from gestational age. Migration status was measured by asking: ‘Where were you born?’ with answer options Sweden (0), Nordic countries but not Sweden (1), Europe but not in the Nordic countries (2), and outside of Europe (3), which was recorded to born in Sweden (0) and born outside of Sweden (1). Relationship status was measured by asking if individuals had a partner (0=no; 1=yes and we live together; 2=yes, but we do not live together), which was recoded into 0=no partner or living apart and 1=living with a partner. Education was measured by asking: ‘What is the highest level of education you have ever completed?’ (0=no schooling, 1=primary school, 2=high school, 3=polytechnic or vocational training, and 4=university or college), which was recoded into 0=no higher education (original score 0–3) and 1=higher education (original score 4). Parity was measured by asking if individuals had more children in their household in addition to their current pregnancy (0=no, 1=yes). History of anxiety disorder was measured by asking individuals: ‘Before pregnancy, have you ever had an anxiety disorder?’ (0=no; 1=yes, and I got help from a psychologist; 2=yes, but I did not seek or receive any help), which was recoded into 0=no and 1=yes, regardless of whether professional help was sought. Finally, the history of pregnancy loss was measured by asking: ‘Have you had a miscarriage or intrauterine fetal death?’ (0=no, 1=yes).

### Statistical analyses

SPSS version 28 was used for all analyses, with a standard threshold of significance at p<0.05 and pairwise deletion for missing values. The specific confounders in the models were decided upon their zero-order correlation with the dependent variable, which was calculated as part of the descriptive analyses. For the descriptive analyses, we calculated the mean, median, and standard deviation for all continuous and ordinal measures, and absolute and relative frequencies of the binary scales.

To test RQ1, we used a hierarchical linear regression model on the cross-sectional dataset with two steps (step 1 = confounders, step 2 = information sources) and PRA as the outcome variable. To test RQ2, we used a multinomial logistic regression to calculate the odds of improving or worsening PRA levels, compared to stable levels. To determine whether PRA was improving, worsening, or remaining stable, we calculated a difference score by subtracting the initial PrAS-score at baseline from the PrAS-score at follow-up. A change of three or more points was considered a deviation, whereby a higher score at follow-up compared to baseline was labeled ‘worsening symptoms’ and a lower score at follow-up compared to baseline was labeled ‘improving symptoms’. The difference score of three points was decided upon discussion with the statistician; probing with different difference scores (1, 5, and 10 points) provided similar results. For both tests, the assumptions were checked and met.

Outside of our hypothesis testing, we explored if the associations between the likelihood of using certain information sources and PRA were modified by parity. This was conducted using four interaction models (one per information source), implemented through Hayes’ PROCESS macro for SPSS, model 1^18^ for the cross-sectional data, and through adding interaction terms to the multinomial logistic regression model for the longitudinal data. Finally, additional exploratory analyses were run to explore whether there would be any differences in our results when looking at specific aspects of PRA instead of the overall anxiety level. To do this, we first ran a multivariate multiple regression in SPSS with the eight subscales as dependent variables, parity and history of anxiety as control variables, and reliance on the midwife, social circle, online sources, and literature for pregnancy and childbirth information as predictor variables in the cross-sectional dataset (RQ1). To examine changes in PRA throughout the pregnancy, we ran separate multinomial logistic regression analyses to calculate the odds of improving or worsening symptoms for each subscale (RQ2). Upon discussion with the statistician, improving symptoms was defined as scoring at least half a point lower, and worsening symptoms was defined as scoring at least half a point higher on the specific subscale at follow-up compared to baseline. Because each subscale had a different number of items, we calculated a mean score per subscale for both analyses.

### Ethical considerations

This study was approved by the Swedish Ethical Review Board in 2019 (initial application DNR: 2019/01170), with an amendment in 2022 to introduce the sub-study. Before entering the study, women had to provide informed consent through the Mom2B app.

## RESULTS

### Descriptive results

**Figure 1:**
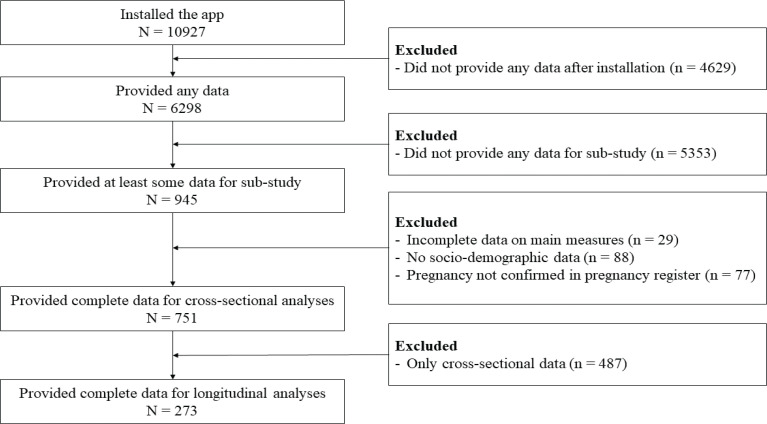
Flowchart of the recruitment and inclusion of participants. Eligibility was checked by confirming the pregnancy against the Swedish pregnancy register. Participants could only participate in the study if they provided data for the sub-study and sociodemographic data (start of study: November 2019; start of sub-study: December 2022)

Figure 1 is the flowchart of the data collection process. Of the 10927 women who installed the app, 6298 provided any research data and 945 provided at least some data for this sub-study. Of those, 751 were included in the cross-sectional dataset and 273 were included in the longitudinal dataset, after confirming their pregnancy in the pregnancy register and ensuring they had provided sociodemographic information, and both PRA and information source data in the same trimester. This means that we had a retention of 36.34% in the longitudinal dataset, compared to the eligible cross-sectional dataset.

Table 1 shows an overview of the participants’ characteristics. Women in our sample had an average PRA score of 57, and approximately one in ten (11.1%) scored above the clinical cut-off score of 75.5. On average, respondents indicated that their midwife was a likely source of pregnancy information, they were undecided about using their social circle or literature, and rather unlikely to use online sources. Most women were born in Sweden, were living with their partner, and had a higher education. Our sample was roughly equally distributed among primiparas and multiparas. Slightly less than half had a history of anxiety and one in three had experienced pregnancy loss in the past.

**Table 1:** Participant characteristics in the Swedish Mom2B sub-study on media use and maternal mental health, December 2022–April 2024

*Characteristics*	*Cross-sectional dataset (N=751)*			*Longitudinal dataset (N=273)*					
				*Baseline*			*Follow-up*		
	*Mean (SD)*	*Median*	*Range*	*Mean (SD)*	*Median*	*Range*	*Mean (SD)*	*Median*	*Range*
**PRA**	57.07 (14.51)	55.00	32–110	57.86 (14.68)	56.00	32–100	56.86 (14.77)	53.00	33–106
**Information sources**									
Midwife	4.13 (0.94)	4.00	1–5	4.08 (0.97)	4.00	1–5	4.27 (1.02)	5.00	1–5
Social circle	3.20 (0.78)	3.20	1–5	3.16 (0.76)	3.20	1–5	3.17 (0.80)	3.20	1–5
Online sources	2.53 (0.60)	2.56	1–5	2.50 (0.60)	2.44	1–4.56	2.52 (0.60)	2.56	1.22–4.22
Literature	3.06 (1.06)	3.00	1–5	3.04 (1.03)	3.00	1–5	3.13 (1.09)	3.00	1–5
**Pregnancy trimester**	1.95 (0.76)	2.00	1–3	1.64 (0.48)	2	1–2	Measured only at baseline		
**Age** (years)	31.88 (4.49)	32	19–44	31.92 (4.51)	31	20–44	Measured only at baseline		
***Personal details***	***n (%)***			***n (%)***					
Born outside Sweden	49 (6.5)			19 (7.0)					
Higher education level	534 (71.1)			200 (73.3)					
Living with partner	705 (93.9)			257 (94.1)					
History of anxiety	332 (44.2)			123 (45.1)					
Multipara	357 (47.5)			127 (46.5)					
History of pregnancy loss	262 (34.9)			89 (32.6)					

PRA: pregnancy-related anxiety (clinical cut-off >75.5).

Of the women who participated in multiple data collections, most entered the study in their second trimester (n=174; 63.7%). Throughout pregnancy, average levels of PRA decreased, though the prevalence of clinical levels of PRA remained relatively stable (13.2% baseline, 12.1% follow-up). When looking at the difference scores, 39.6% (n=108) of the women showed improvement, 28.6% (n=78) had stable PRA levels and 31.9% (n=87) had worsening symptoms (data not shown in table). The likelihood of using the midwife as a source of information increased throughout the pregnancy, whereas the likelihood of using other information sources remained stable.

Table 2 gives the zero-order bivariate correlations between the variables at the cross-sectional level, and Table 3 the zero-order bivariate correlations between the variables at the longitudinal level. Based on the zero-order correlations, only history of anxiety and parity were included as confounders in the cross-sectional model, and baseline PRA and parity in the longitudinal models. When looking at the sources of information, there were positive significant correlations between all sources, except for reliance on the midwife and on online sources, which were not correlated. There was a strong correlation between PRA at baseline, and three months later, indicating a high degree of stability in PRA across time.

**Table 2:** Zero-order bivariate correlations between the variables in the cross-sectional dataset from the Swedish Mom2B sub-study on media use and maternal mental health, December 2022–April 2024 (N=751)

*Variable*	*1*	*2*	*3*	*4*	*5*	*6*	*7*	*8*	*9*	*10*	*11*	*12*
1. PRA												
2. Source: midwife	-0.17***											
3. Source: social circle	-0.07	0.22***										
4. Source: online sources	0.16***	-0.07	0.18***									
5. Source: literature	0.01	0.10**	0.18***	0.24***								
6. Age	-0.04	0.003	-0.16***	-0.12***	-0.01							
7. Pregnancy trimester	-0.03	0.05	0.02	-0.07	-0.04	0.04						
8. Born outside Sweden	0.02	-0.06	-0.12**	0.04	-0.04	0.10**	0.05					
9. Higher education level	-0.04	0.09*	0.02	-0.15***	0.12***	0.16***	-0.01	0.05				
10. Living with partner	-0.06	-0.02	0.01	-0.05	0.001	-0.14*	0.04	0.000	0.06			
11. History of anxiety	0.17***	-0.05	-0.06	0.13***	-0.03	-0.08*	-0.03	0.004	-0.13***	-0.05		
12. Multipara	-0.13***	-0.03	-0.22***	-0.13***	-0.11	0.27***	0.02	-0.01	-0.03	0.08*	-0.05	
13. History of pregnancy loss	-0.01	-0.03	-0.08*	-0.01	-0.03	0.15***	0.05	0.04	-0.09*	0.001	0.02	0.19***

PRA: pregnancy-related anxiety (score: 32–110). Sources measured as the likelihood of using this source for pregnancy- and childbirth-related information (1=extremely unlikely, 5=extremely likely). Variables 8 through 13 are dichotomous variables with 0=no and 1=yes.

* p<0.05

** p<0.01

*** p<0.001.

**Table 3:** Zero-order bivariate correlations between the variable in the longitudinal dataset from the Swedish Mom2B sub-study on media use and maternal mental health, December 2022–April 2024 (N=273)

*Variable*	*1*	*2*	*3*	*4*	*5*	*6*	*7*	*8*	*9*	*10*	*11*	*12*	*13*	*14*
1. PRA at baseline														
2. PRA at follow-up	0.84***													
3. PRA difference score	-0.27***	0.29***												
4. Source: midwife	-0.17**	-0.13*	0.08											
5. Source: social circle	-0.04	0.04	0.14*	0.23***										
6. Source: online sources	0.14*	0.14*	0.004	-0.01	0.21***									
7. Source: literature	-0.08	0.004	0.14*	0.16**	0.22***	0.21***								
8. Age	-0.07	-0.08	-0.02	-0.06	-0.21***	-0.06	-0.10							
9. Pregnancy trimester	0.01	0.01	0.02	-0.04	0.06	-0.13*	-0.10	0.09						
10. Born outside Sweden	0.01	-0.04	-0.09	-0.05	-0.10	0.01	-0.07	0.14*	-0.003					
11. Higher education	-0.11	-0.11	-0.002	-0.03	-0.02	-0.20***	0.15*	0.18**	-0.03	0.07				
12. Living with partner	-0.06	-0.05	0.01	-0.04	0.03	-0.17**	-0.01	-0.15*	-0.06	0.01	0.13*			
13. History of anxiety	0.17**	0.17**	-0.01	-0.03	0.03	0.19**	-0.06	-0.04	-0.05	0.01	-0.22***	-0.12*		
14. Multipara	-0.23***	-0.13*	0.17**	-0.03	-0.26***	-0.07	-0.11	0.29***	0.02	0.09	-0.001	0.05	-0.03	
15. History of pregnancy loss	0.07	0.10	0.06	-0.02	-0.05	-0.04	-0.06	0.14*	0.10	0.03	-0.07	0.01	0.03	0.20***

PRA: pregnancy-related anxiety. Except for PRA at follow-up and PRA difference score, all variables are measured at baseline. Sources measured as the likelihood of using this source for pregnancy- and childbirth-related information (1=extremely unlikely, 5=extremely likely). Variables 10 through 15 are dichotomous variables with 0=no and 1=yes.

* p<0.05

** p<0.01

*** p<0.001.

### RQ1: The association between sources of pregnancy information and concurrent PRA

First, we tested the cross-sectional association between the likelihood of using different sources of pregnancy- and childbirth-related information and the level of PRA (Table 4). We found that stronger reliance on their midwife related to lower levels of PRA. In contrast, stronger reliance on online sources related to higher levels of PRA. Women who were more likely to rely on their social circle also reported lower levels of PRA, but only when entering parity as a confounder. When looking at the control variables, primiparas (B= -3.75, SE=1.05, t= -3.58, p<0.001) and women with a history of anxiety (B=3.93, SE=1.04, t=3.80, p<0.001) also had higher levels of PRA. Additional exploratory moderation analyses indicated that parity did not moderate the associations between the likelihood of relying on the midwife (B=1.06, SE=1.09, t=0.97, p=0.33), social circle (B=0.32, SE=1.35, t=0.24, p=0.81), online sources (B= -0.80, SE=1.71, t= -0.47, p=0.64) or literature (B=0.28, SE=0.97, t=0.29, p=0.77) for information about pregnancy and childbirth and PRA (data not shown in Table form).

**Table 4:** The association between the likelihood of using certain sources of pregnancy information and pregnancy-related anxiety (RQ1) using the crosssectional data from the Swedish Mom2B sub-study on media use and maternal mental health, December 2022–April 2024 (N=751)

*Variable*	*Unadjusted model*				*Confounder-adjusted model*				*Fully adjusted model*			
	*B (SE)*	*β*	*t*	*95% CI*	*B (SE)*	*β*	*t*	*95% CI*	*B (SE)*	*Β*	*t*	*95% CI*
Midwife	-2.68 (0.56)***	-0.17	-4.82	-3.77 – -1.59	-2.63 (0.55)***	-0.17	-4.82	-3.70 – -1.56	-2.23 (0.56)***	-0.14	-3.98	3.32 – -1.13
Social circle	-1.22 (0.68)	-0.07	-1.79	-2.55–0.12	-1.64 (0.68)*	-0.09	-2.40	-2.99 – -0.30	-1.45 (0.70)*	-0.08	-2.07	-2.83 – -0.07
Online sources	3.94 (0.87)***	0.16	4.54	2.24–5.65	3.17 (0.87)***	0.13	3.65	1.46–4.87	3.28 (0.89)***	0.14	3.67	1.52–5.03
Literature	0.06 (0.50)	0.01	0.13	-0.92–1.05	-0.06 (0.50)	-0.004	-0.12	-1.03–0.92	-0.12 (0.50)	-0.01	-0.24	-1.11–0.87

Unadjusted model: simple linear regression without covariates. Confounder-adjusted model: linear regression controlling for history of anxiety and parity. Fully adjusted model: linear regression controlling for all variables in the confounderadjusted model plus the other sources of pregnancy-information.

* p<0.05

** p<0.01

*** p<0.001.

When examining the subscales of PRA (see the Supplementary file for the full results), we found that the multivariate test was significant for reliance on the midwife [Pillai’s trace=0.04, F(8,737)=3.65, p<0.001, η_p_^²^=0.04] and reliance on online sources [Pillai’s trace=0.03, F(8,737)=2.48, p<0.05, η_p_^²^=0.03]. The tests of betweensubjects revealed significant negative associations between reliance on the midwife and significant positive associations between reliance on online sources, and all PRA subscales, except for avoidance of a vaginal birth and acceptance of the pregnancy. In contrast, the multivariate tests were non-significant for reliance on the social circle [Pillai’s trace=0.01, F(8,737)=1.14, p=0.34, η_p_²=0.01) or literature (Pillai’s trace=0.02, F(8,737)=1.83, p=0.07, η_p_²=0.02]. However, the test of between-subject effects did reveal some significant associations with individual dependent variables. Specifically, reliance on the social circle was associated with less body image concerns and more acceptance of the pregnancy, and reliance on literature was associated with less body image concerns.

### RQ2: The association between sources of pregnancy information and changes in PRA throughout the pregnancy

Second, we tested whether the likelihood of using different sources of pregnancy- and childbirth-related information could predict the likelihood of improving or worsening symptoms of PRA in the consecutive pregnancy trimester (Table 5). When controlling for parity and baseline level of PRA, we only found that relying more strongly on literature for information was associated with lower odds of improvement of PRA. No other information sources predicted changes in PRA throughout the pregnancy. Regarding the control variables, women with higher levels of PRA at baseline had slightly higher odds to improve over time (OR=1.05, p<0.001) but not to worsen (OR=1.02, p=0.15) and parity predicted neither improving (OR=-0.43, p=0.21) nor worsening (OR=0.59, p=0.08) PRA symptoms. Additional exploratory moderation analyses indicated that parity did not moderate the associations between the likelihood of using the midwife (χ^²^=0.42, p=0.81), social circle (χ^²^=1.65, p=0.44), online sources (χ^²^=1.83, p=0.40) or literature (χ^²^=1.44, p=0.49) as a source of information in predicting PRA changes (data not shown in Table form).

**Table 5:** The association between sources of pregnancy information and changes in PRA throughout the pregnancy (RQ2) using the longitudinal data from the Swedish Mom2B sub-study on media use and maternal mental health, December 2022–April 2024 (N=273)

*Variable*	*Unadjusted models*				*Confounder-adjusted models*				*Fully adjusted model*			
	*B (SE)*	*Wald*	*OR*	*95% CI*	*B (SE)*	*Wald*	*OR*	*95% CI*	*B (SE)*	*Wald*	*OR*	*95% CI*
	*Odds of improving PRA symptoms*											
Midwife	-0.07 (0.15)	0.24	0.93	0.69–1.25	0.03 (0.16)	0.03	1.03	0.75–1.41	0.09 (0.17)	0.29	1.09	0.79–1.52
Social circle	-0.04 (0.19)	0.04	0.96	0.66–1.41	-0.10 (0.21)	0.22	0.91	0.60–1.37	0.02 (0.23)	0.01	1.02	0.65–1.60
Online sources	-0.12 (0.25)	0.25	0.88	0.54–1.44	-0.32 (0.27)	1.49	0.72	0.43–1.22	-0.18 (0.28)	0.40	0.84	0.49–1.45
Literature	-0.47 (0.15)**	9.47	0.63	0.46–0.84	-0.50 (0.16)**	9.41	0.61	0.45–0.84	-0.49 (0.17)**	8.55	0.62	0.45–0.85
***Odds of worsening PRA symptoms***												
Midwife	0.07 (0.17)	0.17	1.07	0.77–1.48	0.12 (0.17)	0.46	1.12	0.80–1.57	0.05 (0.17)	0.09	1.06	0.75–1.49
Social circle	0.27 (0.21)	1.63	1.31	0.87–1.96	0.37 (0.22)	2.90	1.44	0.95–2.20	0.40 (0.23)	3.13	1.50	0.96–2.34
Online sources	-0.10 (0.26)	0.14	0.91	0.54–1.51	-0.12 (0.26)	0.21	0.89	0.53–1.48	-0.22 (0.28)	0.60	0.81	0.47–1.40
Literature	-0.06 (0.16)	0.15	0.94	0.69–1.28	-0.03 (0.16)	0.03	0.97	0.71–1.33	-0.05 (0.17)	0.10	0.95	0.68–1.32

PRA: pregnancy-related anxiety. Improving symptoms is defined as scoring at least 3 points lower and worsening symptoms is defined as scoring at least 3 points higher on the PrAS at follow-up compared to baseline. Unadjusted model: multinomial logistic regression without covariates. Confounder-adjusted model: multinomial logistic regression controlling for PRA at baseline and parity. Fully adjusted model: multinomial logistic regression controlling for all variables in the confounder-adjusted model plus the other information sources.

* p<0.05

** p<0.01

*** p<0.001.

When examining changes in subscales of PRA throughout the pregnancy (see the Supplementary file for full results), we found that the more likely women were to use their midwife as a source of information, the less likely they were to have increasingly negative attitudes towards healthcare providers throughout the pregnancy (OR=0.65, p=0.02). The more likely women were to rely on their social circle for information, the less likely they were to improve their attitude towards childbirth throughout the pregnancy (OR=0.64, p=0.046). Women who were more likely to use online information sources were almost twice as likely to improve their concerns about their baby’s wellbeing throughout the pregnancy (OR=1.89, p=0.04). Finally, the more likely women were to use literature for information, the less likely they were to improve their self-worry (OR=0.61, p=0.049) and the more likely they were to have increasingly negative attitudes towards healthcare providers (OR=1.46, p=0.046).

## DISCUSSION

The aim of this study was to understand to what extent different information sources are associated with pregnancy-related anxiety (PRA), and also which are helpful to women for reducing PRA. To date, very little research has been conducted on the associations between PRA and information seeking. This is the first time that the relation between four different sources of information and PRA are compared, and their longitudinal impact on PRA fluctuations are studied.

In line with our expectations and prior research, we found that – at the cross-sectional level – greater reliance on the midwife as an information source was associated with lower levels of PRA, whereas greater reliance on online sources was associated with higher levels of PRA. This coincides with prior findings on the positive association between engagement with ‘mommy influencers’ and motherhood-anxiety^8^, media users’ qualitative experiences of increased anxiety when finding it hard to stop searching the web for information^7^, and greater anxiety among pregnant women undergoing invasive prenatal testing when relying on media sources^11^, though one other study found that online information searching reduced anxiety among women hospitalized for pregnancy complications^10^. In line with one study on anxiety among pregnant women undergoing invasive prenatal testing^11^, greater reliance on the social circle was also associated with less PRA. However, in our study, reported use of these sources did not predict changes in PRA over time. Only greater reliance on (e-)books was associated with a lower likelihood of improving PRA symptoms over time, even though it was not associated with PRA at the cross-sectional level. Based on these results, we can draw several conclusions on the helpfulness of different information sources in reducing PRA.

First, building on the cross-sectional results, it seems that especially interpersonal sources (i.e. the midwife and social circle) are helpful in mitigating PRA, though causality cannot be established. A possible explanation for this, are the direct interactions between anxious women and interpersonal information sources. If women voice their fears, midwives or friends can immediately react and provide social support or trustworthy information (especially midwives) to explain why certain fears are unlikely to occur. This interpersonal back-and-forth of concerns and help may result in lower levels of PRA. In contrast, online sources rely predominantly on asynchronous or one-directional (sender to receiver) communication. Interactions are non-existent (e.g. on government websites) or delayed (e.g. on forums), and prior research has shown that online pregnancy and postpartum forums are often filled with drama, ‘trolls’, and belittling comments from others when asking questions or voicing struggles^19^. Furthermore, the reliability of online information is often questionable^4-6^ and women can struggle with stopping their search when it is no longer helpful^7^, potentially resulting in information overload. Taken together, this may result in less social support and increased anxiety when relying on online sources for information.

However, these relationships were not found when looking at the longitudinal data. Four potential explanations could help us understand why reliance on different information sources did not predict deviations in PRA levels throughout the pregnancy. First, it is possible that the timespan between our measurements was too long. In this study, we measured PRA and information sources once per pregnancy trimester, resulting in approximately three months between measurements. However, it is possible that information seeking behaviors affect PRA levels in a much shorter time span. To test this, we recommend future research to either design longitudinal studies with a shorter time lag (e.g. 2 weeks to a month), or to use experience sampling methodology to model daily fluctuations between information sources and PRA levels. This will provide a better understanding of whether information seeking really affects PRA within a shorter timeframe, or not at all.

Second, it is possible that the relationship between PRA and information seeking is misunderstood, and it is really PRA driving information seeking instead of vice versa. Thus, this would mean that PRA predicts which information sources women rely on. However, while we believe this is a possible explanation for the association between PRA and online information seeking (higher PRA results in more reliance on online sources), we do not believe that this reflects the true nature between PRA and offline information seeking (higher PRA results in less reliance on the midwife for information). Furthermore, when running an exploratory reverse analysis, no significant associations between baseline PRA and reliance on any of the information sources at follow-up was found. We thus do not believe that this explains our results.

Third, it could also be that our findings reflect differences between groups of women, rather than changes within individual women over time as a result of reliance on certain information sources. In other words, the results might indicate that, on average, women who are generally more anxious are more likely to rely on online information and less likely to rely on their midwife or social circle compared to women who are less anxious. This would suggest a between-person effect, where more anxious women as a group differ in their information-seeking behaviors from less anxious women. However, this does not necessarily imply a within-person effect, where an individual woman’s increased reliance on online information would lead to higher levels of PRA, or that her reliance on her midwife or social circle would reduce her PRA. Instead, our results could be interpreted as showing that women who are already more anxious tend to seek additional information online more than those who are less anxious, but this behavior does not necessarily cause changes in their own levels of PRA. The lower reliance on the midwife could potentially be explained because more anxious women do not trust that their midwife has their best interest at heart, or because they feel not listened to when voicing their anxieties. This interpretation would explain the difference between our cross-sectional and longitudinal results. Future research could formally test this hypothesis by using multilevel models that differentiate between between-person effects and within-person effects^20-22^.

Finally, it is possible that the likelihood of relying on certain sources of information is less important than the frequency with which these sources are consulted, if we want to understand their impact on PRA. Anxious women who believe their midwife is the most reliable source of information and report that this is their most likely source of information, would not reap the benefits of these beliefs if they do not actually reach out to her whenever needed, or voice their concerns during a booked consultation. Similarly, reliance on online information is likely not resulting in detrimental PRA levels if a woman looks up information only once in a while, despite reporting that this is their most likely source of information. Future research needs to incorporate not only the likelihood of relying on certain sources but also the frequency of consulting these sources to better understand how different information sources can affect PRA. Qualitative research approaches are also important in this field.

### Strengths and limitations

Our study has several strengths. First, we combined cross-sectional and longitudinal data to obtain the fullest picture of the associations between reliance on different information sources and PRA. The study was nested within a population-based national sample of pregnant women in Sweden, using a modern tool for user-friendly data collection^12^. We also had access to several possible confounding factors at the individual level and were able to appropriately adjust our statistical analyses.

The study has some limitations. It needs to be noted that Mom2B participants are overall more highly educated and Swedish-born than the general population of pregnant women in Sweden; results can therefore not be readily generalizable. Further, data collection was more difficult than anticipated, resulting in lower retention than expected, and thus a relatively small sample size for the longitudinal analyses, albeit in line with other published studies to date^23-25^. Our longitudinal model is likely underpowered and should be interpreted with caution. Moreover, attrition bias may play a role in who is most likely to drop out of research. Finally, this study was conducted in Sweden, a rich country with universal healthcare and a midwifery-led model of care. The results are potentially not generalizable to other societies.

## CONCLUSIONS

Not all information sources play an equal role in relation to PRA. Whereas reliance on healthcare professionals or the social circle was associated with lower levels of PRA, reliance on online information correlated with higher levels of PRA. However, reliance on the different information sources did not predict changes in PRA in a consecutive pregnancy trimester. More research in bigger samples, with more dimensions of information-seeking behavior, is needed to understand whether it is indeed unrelated to PRA fluctuations.

## Supplementary Material



## Data Availability

The data supporting this research are available from the authors on reasonable request.

## References

[1] Lu Y, Barrett LA, Lin RZ, Amith M, Tao C, He Z. Understanding information needs and barriers to accessing health information across all stages of pregnancy: systematic review. JMIR Pediatr Parent. 2022;5(1):e32235. doi:10.2196/32235 35188477 PMC8902674

[2] Ghiasi A. Health information needs, sources of information, and barriers to accessing health information among pregnant women: a systematic review of research. J Matern Fetal Neonatal Med. 2021;34(8):1320-1330. doi:10.1080/14767058.2019.1634685 31216921

[3] Lanssens D, Thijs IM, Dreesen P, et al. Information resources among Flemish pregnant women: crosssectional study. JMIR Form Res. 2022;6(10):e37866. doi:10.2196/37866 36222794 PMC9597425

[4] Conrad M. Health information-seeking internet behaviours among pregnant women: a narrative literature review. J Reprod Infant Psychol. 2024;42(2):194-208. doi:10.1080/02646838.2022.2088711 35703164

[5] D’Souza RS, D’Souza S, Sharpe EE. YouTube as a source of medical information about epidural analgesia for labor pain. Int J Obstet Anesth. 2021;45:133-137. doi:10.1016/j.ijoa.2020.11.005 33339713

[6] Hansen C, Interrante JD, Ailes EC, et al. Assessment of YouTube videos as a source of information on medication use in pregnancy. Pharmacoepidemiol Drug Saf. 2016;25(1):35-44. doi:10.1002/pds.3911 26541372 PMC4707975

[7] Prescott J, Mackie L. “You sort of go down a rabbit hole… You’re just going to keep on searching”: a qualitative study of searching online for pregnancy-related information during pregnancy. J Med Internet Res. 2017;19(6):e194. doi:10.2196/jmir.6302 28583906 PMC5476867

[8] Moujaes M, Verrier D. Instagram use, instamums, and anxiety in mothers of young children. J Media Psychol. 2021;33(2):72-81. doi:10.1027/1864-1105/a000282

[9] Šoštarić M, Jokić-Begić N, Vukušić Mijačika M. Can›t stop, won›t stop - understanding anxiety›s role in cyberchondria among pregnant women. Women Health. 2024;64(2):185-194. doi:10.1080/03630242.2024.2308525 38258443

[10] Coglianese F, Beltrame Vriz G, Soriani N, et al. Effect of online health information seeking on anxiety in hospitalized pregnant women: cohort study. JMIR Med Inform. 2020;8(5):e16793. doi:10.2196/16793 32374268 PMC7240442

[11] Çakar M, Tari Kasnakoglu B, Ökem ZG, Okuducu Ü, Beksaç MS. The effect of different information sources on the anxiety level of pregnant women who underwent invasive prenatal testing. J Matern Fetal Neonatal Med. 2016;29(23):3843-3847. doi:10.3109/14767058.2016.1149560 26867089

[12] Bilal AM, Fransson E, Bränn E, et al. Predicting perinatal health outcomes using smartphone-based digital phenotyping and machine learning in a prospective Swedish cohort (Mom2B): study protocol. BMJ Open. 2022;12(4):e059033. doi:10.1136/bmjopen-2021-059033 PMC9047888 35477874

[13] Bilal AM, Pagoni K, Iliadis SI, Papadopoulos FC, Skalkidou A, Öster C. Exploring user experiences of the Mom2B mHealth research app during the perinatal period: qualitative study. JMIR Form Res. 2024;8:e53508. doi:10.2196/53508 39115893 PMC11342009

[14] Zhong M, Van Zoest V, Bilal AM, Papadopoulos F, Castellano G. Unimodal vs. multimodal prediction of antenatal depression from smartphone-based survey data in a longitudinal study. Paper presented at: 2022 International Conference on Multimodal Interaction; 2022. ACM; 2022:455-467. doi:10.1145/3536221.3556605

[15] Fransson E, Karalexi M, Kimmel M, et al. Mental health among pregnant women during the pandemic in Sweden–a mixed methods approach using data from the Mom2B mobile application for research. medRxiv. Preprint posted online December 20, 2020. doi:10.1101/2020.12.18.20248466

[16] Brunton RJ, Dryer R, Saliba A, Kohlhoff J. The initial development of the pregnancy-related anxiety scale. Women Birth. 2019;32(1):e118-e130. doi:10.1016/j.wombi.2018.05.004 29859678

[17] Brunton R, Dryer R, Saliba A, Kohlhoff J. Re-examining pregnancy-related anxiety: a replication study. Women Birth. 2019;32(1):e131-e137. doi:10.1016/j.wombi.2018.04.013 29747955

[18] Hayes AF. Introduction to Mediation, Moderation, and Conditional Process Analysis: A Regression-Based Approach. The Guilford Press; 2013.

[19] Teaford D, McNiesh S, Goyal D. New mothers’ experiences with online postpartum forums. MCN Am J Matern Child Nurs. 2019;44(1):40-45. doi:10.1097/NMC.0000000000000489 30444739

[20] Hamaker EL, Kuiper RM, Grasman RP. A critique of the cross-lagged panel model. Psychol Methods. 2015;20(1):102-116. doi:10.1037/a0038889 25822208

[21] Curran PJ, Bauer DJ. The disaggregation of within-person and between-person effects in longitudinal models of change. Annu Rev Psychol. 2011;62:583-619. doi:10.1146/annurev.psych.093008.100356 19575624 PMC3059070

[22] Curran PJ, Howard AL, Bainter SA, Lane ST, McGinley JS. The separation of between-person and within-person components of individual change over time: a latent curve model with structured residuals. J Consult Clin Psychol. 2014;82(5):879-894. doi:10.1037/a0035297 24364798 PMC4067471

[23] Wang MJ, Dunn EC, Okereke OI, Kraft P, Zhu Y, Smoller JW. Maternal vitamin D status during pregnancy and offspring risk of childhood/adolescent depression: results from the Avon Longitudinal Study of Parents and Children (ALSPAC). J Affect Disord. 2020;265:255-262. doi:10.1016/j.jad.2020.01.005 32090749 PMC7448808

[24] Yun I, Jung YH, Park EC, Jang SI. The impact of work interference with family on depressive symptoms among married working women: a longitudinal panel study. PLoS One. 2022;17(11):e0276230. doi:10.1371/journal.pone.0276230 36350817 PMC9645602

[25] Geusens F, Van Uytsel H, Ameye L, et al. The impact of self-monitoring physical and mental health via an mHealth application on postpartum weight retention: data from the INTER-ACT RCT. Health Promot Perspect. 2024;14(1):44-52. doi:10.34172/hpp.42528 38623343 PMC11016147

