# Supplementary material for: A two-wave survey study examining the impact of different sources of pregnancy information on pregnancy-related anxiety among Swedish women
Source: Eur J Midwifery. 2025 Jan 17;9:10.18332/ejm/197169. doi: 10.18332/ejm/197169 (PMC11739933; doi:10.18332/ejm/197169)
Supplement: Supplementary file 1 [file EJM-9-06-s1.pdf]

Supplementary file: Additional analyses exploring the different anxiety subscales.

Table 1. The associations between using different sources of pregnancy information and different aspects of pregnancy-related anxiety (RQ1)

Variable	B(SE)	t	η_p^2	95% CI
**Dependent variable 1: childbirth concerns** (adjusted R^2 = .05)				
Intercept	1.87(.19)***	10.04	.12	[1.50; 2.23]
History of anxiety	.11(.05)*	2.14	.01	[.01; .21]
Multipara	-.22(.05)***	-4.09	.02	[-.32; -.11]
Midwife as information source	-.07(.03)*	-2.42	.01	[-.12; -.01]
Social circle as information source	-.02(.04)	-.50	.000	[-.09; .05]
Online sources as information source	.13(.05)**	2.85	.01	[.04; .22]
Literature as information source	.03(.03)	1.16	.002	[-.02; .08]
**Dependent variable 2: body image concerns** (adjusted R^2 = .05)				
Intercept	2.30(.20)***	11.73	.16	[1.91; 2.68]
History of anxiety	.18(.06) ***	3.32	.02	[.07;.29]
Multipara	.06(.06)	1.04	.001	[-.05;.17]
Midwife as information source	-.09(.03)**	-2.90	.01	[-.14; -.03]
Social circle as information source	-.07(.04)	-1.96	.01	[-.15; .000]
Online sources as information source	.11(.05)*	2.33	.01	[.02; .20]
Literature as information source	-.06(.03)*	-2.20	.01	[-.11; -.01]
**Dependent variable 3: attitudes towards childbirth** (adjusted R^2 = .09)				
Intercept	2.80(.22)***	12.94	.18	[2.37; 3.22]
History of anxiety	.07(.06)	1.08	.002	[-.05; .18]
Multipara	-.46(.06)***	-7.58	.07	[-.58; -.34]
Midwife as information source	-.07(.03)*	-2.12	.01	[-.13; -.01]
Social circle as information source	-.04(.04)	-.93	.001	[-.12; .04]
Online sources as information source	.12(.05)*	2.29	.01	[.02; .22]
Literature as information source	-.01(.03)	-.28	.000	[-.07; .05]
**Dependent variable 4: worry about self** (adjusted R^2 = .07)				
Intercept	1.53(.13)***	11.59	.15	[1.27; 1.79]
History of anxiety	.21(.04)***	5.83	.04	[.14; .29]
Multipara	-.06(.04)	-1.54	.003	[-.13; .02]
Midwife as information source	-.04(.02)*	-2.09	.01	[-.08; -.002]
Social circle as information source	-.04(.03)	-1.74	.004	[-.09; .01]
Online sources as information source	.09(.03)**	2.96	.01	[.03; .16]
Literature as information source	-.002(.02)	-.12	.000	[-.04; .03]
**Dependent variable 5: baby concerns** (adjusted R^2 = .06)				
Intercept	2.37(.21)***	11.46	.15	[1.97; 2.78]
History of anxiety	.11(.06)	1.96	.01	[.000; .23]
Multipara	-.24(.06)***	-4.10	.02	[-.35; -.13]
Midwife as information source	-.10(.03)***	-3.28	.01	[-.16; -.04]
Social circle as information source	-.06(.04)	-1.48	.003	[-.13; .02]

Online sources as information source	.16(.05)***	3.22	.01	[.06; .26]
Literature as information source	-.03(.03)	-.93	.001	[-.08; .03]
**Dependent variable 6: non-acceptance of pregnancy** (adjusted $R^2 = .01$)				
Intercept	1.50(.14)***	10.98	.14	[1.23; 1.77]
History of anxiety	-.02(.04)	-.58	.000	[-.10; .05]
Multipara	.06(.04)	1.53	.003	[-.02; .13]
Midwife as information source	-.003(.02)	-.15	.000	[-.04; .04]
Social circle as information source	-.06(.03)*	-2.28	.01	[-.11; -.01]
Online sources as information source	.03(.03)	.90	.001	[-.04; .09]
Literature as information source	-.02(.02)	-.99	.001	[-.06; .02]
**Dependent variable 7: avoidance of vaginal birth** (adjusted $R^2 = .001$)				
Intercept	1.84(.21)***	8.73	.09	[1.42; 2.25]
History of anxiety	.03(.06)	.53	.000	[-.08; .15]
Multipara	-.04(.06)	-.74	.001	[-.16; .07]
Midwife as information source	-.06(.03)	-1.92	.01	[-.12; .001]
Social circle as information source	-.04(.04)	-1.06	.002	[-.12; .04]
Online sources as information source	.01(.05)	.22	.000	[-.09; .11]
Literature as information source	.004(.03)	.15	.000	[-.05; .06]
**Dependent variable 8: attitudes towards medical staff** (adjusted $R^2 = .07$)				
Intercept	1.88(.21)***	9.17	.10	[1.48; 2.29]
History of anxiety	.17(.06)**	3.00	.01	[.06; .28]
Multipara	-.12(.06)	-1.99	.01	[-.23; -.002]
Midwife as information source	-.15(.03)***	-4.74	.03	[-.21; -.09]
Social circle as information source	-.05(.04)	-1.15	.002	[-.12; .03]
Online sources as information source	.15(.05)**	2.97	.01	[.05; .24]
Literature as information source	.05(.03)	1.85	.01	[-.003; .11]

Notes: * $p < .05$, ** $p < .01$, *** $p \leq .001$

Table 2. The association between different sources of pregnancy information and changes in different aspects of pregnancy-related anxiety throughout the pregnancy (RQ2)

	Odds of improving childbirth concerns				Odds of worsening childbirth concerns			
Variable	B(SE)	Wald	OR	95% CI OR	B(SE)	Wald	OR	95% CI OR
Intercept	-3.48(1.57)*	4.90			-1.79(1.24)	2.07		
Childbirth concerns at baseline	1.79(.32)***	32.03	5.96	[3.21; 11.06]	.17(.24)	.48	1.18	[.74; 1.89]
Multipara (0 = no, 1 = yes)	-.70(.49)	2.01	.50	[.19; 1.30]	.27(.33)	.66	1.31	[.68; 2.51]
Midwife	.27(.23)	1.35	1.31	[.83; 2.05]	-.04(.17)	.06	.96	[.69; 1.34]
Social circle	-.19(.33)	.32	.83	[.44; 1.59]	.27(.22)	1.48	1.31	[.85; 2.03]
Online sources	-.52(.38)	1.85	.60	[.28; 1.26]	-.24(.28)	.75	.78	[.45; 1.36]
Literature	-.41(.22)	3.39	.66	[.43; 1.03]	.01(.16)	.003	1.01	[.74; 1.39]
	Odds of improving body image concerns				Odds of worsening body image concerns			
Variable	B(SE)	Wald	OR	95% CI OR	B(SE)	Wald	OR	95% CI OR
Intercept	-2.64(1.43)	3.39			-3.66(1.53)*	5.73		
Body image concerns at baseline	1.35(.25)***	29.07	3.87	[2.37; 6.32]	.29(.28)	1.05	1.33	[.77; 2.31]
Multipara (0 = no, 1 = yes)	-.51(.42)	1.48	.60	[.27; 1.36]	.05(.39)	.02	1.05	[.49; 2.26]
Midwife	.10(.21)	.24	1.11	[.73; 1.68]	.01(.21)	.003	1.01	[.67; 1.53]
Social circle	-.27(.30)	.82	.76	[.43; 1.37]	.53(. 29)	3.47	1.70	[.97; 2.99]
Online sources	-.32(.37)	.78	.72	[.35; 1.48]	.05(.33)	.02	1.05	[.55; 2.01]
Literature	-.17(.19)	.77	.85	[.58; 1.23]	-.21(.20)	1.11	.81	[.55; 1.20]

	Odds of improving attitudes towards childbirth				Odds of worsening attitudes towards childbirth			
Variable	B(SE)	Wald	OR	95% CI OR	B(SE)	Wald	OR	95% CI OR
Intercept	-.89(1.24)	.52			-2.05(1.59)	1.68		
Attitudes towards childbirth at baseline	.67(.20)***	11.86	1.96	[1.34; 2.88]	-.47(.26)	3.32	.62	[.38; 1.04]
Multipara (0 = no, 1 = yes)	-.50(.35)	2.04	.67	[.31; 1.20]	.32(.46)	.48	1.38	[.56; 3.40]
Midwife	.11(.16)	.46	1.12	[.81; 1.54]	.08(.23)	.12	1.08	[.69; 1.70]
Social circle	-.45(.23)*	3.98	.64	[.41; .99]	-.16(.29)	.31	.85	[.48; 1.51]
Online sources	-.13(.27)	.23	.88	[.52; 1.49]	.39(.35)	1.25	1.47	[.75; 2.89]
Literature	-.18(.16)	1.24	.84	[.61; 1.14]	.12(.21)	.32	1.13	[.75; 1.69]
	Odds of improving worry about self				Odds of worsening worry about self			
Variable	B(SE)	Wald	OR	95% CI OR	B(SE)	Wald	OR	95% CI OR
Intercept	-5.92(1.95)**	9.20			-3.82(1.41)**	7.30		
Worry about self at baseline	2.83(.48)*** .	34.91	16.90	[6.62; 43.19]	1.00(.42)*	5.80	2.73	[1.21; 6.17]
Multipara (0 = no, 1 = yes)	22(.53) .48(.3	.17	1.24	[.44; 3.52]	.56(.38)	2.23	1.76	[.84; 3.69]
Midwife	1)	2.35	1.62	[.88; 2.99]	-.26(.18)	2.11	.77	[.54; 1.10]
Social circle	-.36(.38)	.88	.70	[.33; 1.48]	.29(.27)	1.15	1.33	[.79; 2.24]
Online sources	-.22(.43)	.26	.80	[.34; 1.88]	.05(.32)	.02	1.05	[.56; 1.95]
Literature	-.49(.25)*	3.89	.61	[.37; .997]	.13(.19)	.46	1.13	[.79; 1.63]
	Odds of improving baby concerns				Odds of worsening baby concerns			
Variable	B(SE)	Wald	OR	95% CI OR	B(SE)	Wald	OR	95% CI OR
Intercept	-6.27(1.45)***	18.67			-4.88(1.56)**	9.82		
Baby concerns at baseline	1.67(.25)***	44.62	5.29	[3.24; 8.62]	.23(.29)	.66	1.26	[.72; 2.22]
Multipara (0 = no, 1 = yes)	.59(.37)	2.53	1.80	[.87; 3.70]	.64(.38)	2.76	1.89	[.89; 3.99]
Midwife	.01(.18)	.004	1.01	[.71; 1.45]	.22(.23)	.93	1.25	[.80; 1.96]
Social circle	.39(.26)	2.26	1.48	[.89: 2.47]	.32(.27)	1.39	1.37	[.81; 2.33]
Online sources	.63(.30)*	4.46	1.89	[1.05; 3.39]	.50(.31)	2.58	1.65	[.90; 3.04]
Literature	-.54(.18)**	8.93	.58	[.41; .83]	-.19(.19)	.93	.83	[.57; 1.21]

	Odds of improving non-acceptance of pregnancy				Odds of worsening non-acceptance of pregnancy			
Variable	B(SE)	Wald	OR	95% CI OR	B(SE)	Wald	OR	95% CI OR
Intercept	-4.70(1.98)*	5.63			-.79(1.76)	.20		
Non-acceptance at baseline	2.68(.44)***	36.51	14.54	[6.10; 34.66]	.49(.52)	.90	1.63	[.59; 4.47]
Multipara (0 = no, 1 = yes)	.12(.55)	.05	1.13	[.39; 3.29]	-.47(.52)	.80	.63	[.23; 1.74]
Midwife	-.48(.27)	3.21	.62	[.37; 1.05]	-.41(.23)	3.22	.67	[.43; 1.04]
Social circle	.12(.41)	.08	1.12	[.50; 2.50]	.01(.35)	.001	1.01	[.51; 2.03]
Online sources	.03(.48)	.003	1.03	[.40; 2.64]	.03(.43)	.004	1.03	[.44; 2.40]
Literature	-.15(.27)	.32	.86	[.51; 1.45]	-.21(.24)	.77	.81	[.50; 1.30]
	Odds of vaginal birth avoidance				Odds of worsening vaginal birth avoidance			
Variable	B(SE)	Wald	OR	95% CI OR	B(SE)	Wald	OR	95% CI OR
Intercept	-2.92(1.53)	3.66			-4.42(2.04)*	4.70		
Avoidance at baseline	1.34(.22)***	37.85	3.81	[2.49; 5.83]	.63(.30)*	4.23	1.87	[1.03; 3.39]
Multipara (0 = no, 1 = yes)	-1.35(.51)**	7.12	.26	[.10; .70]	.01(.56)	.001	1.01	[.34; 3.01]
Midwife	.18(.24)	.57	1.20	[.75; 1.93]	.003(.30)	.000	1.003	[.56; 1.80]
Social circle	-.52(.33)	2.49	.60	[.31; 1.13]	.41(.41)	.99	1.50	[.68; 3.34]
Online sources	-.08(.36)	.05	.93	[.46; 1.87]	.04(.47)	.01	1.04	[.42; 2.60]
Literature	.06(.22)	.07	1.06	[.69; 1.62]	-.23(.28)	.64	.80	[.46; 1.39]
	Odds of improving attitudes towards medical staff				Odds of worsening attitudes towards medical staff			
Variable	B(SE)	Wald	OR	95% CI OR	B(SE)	Wald	OR	95% CI OR
Intercept	-2.14(1.33)	2.59			-.04(1.28)	.001		
Attitudes towards medical staff at baseline	1.28(.21)***	36.41	3.60	[2.38; 5.46]	-.36(.28)	1.75	.70	[.41; 1.19]
Multipara (0 = no, 1 = yes)	-.78(.40)	3.80	.46	[.21; 1.00]	-.44(.37)	1.37	.65	[.31; 1.34]
Midwife	.13(.19)	.43	1.13	[.78; 1.65]	-.43(.18)*	5.80	.65	[.46; .92]
Social circle	-.44(.27)	2.63	.65	[.38; 1.10]	-.08(.25)	.10	.92	[.56; 1.52]
Online sources	-.07(.31)	.04	.94	[.51; 1.73]	.05(.33)	.03	1.06	[.56; 1.99]
Literature	-.13(.18)	.48	.88	[.62; 1.26]	.38(.19)*	4.00	1.46	[1.01; 2.10]

Notes: * $p < .05$, ** $p < .01$, *** $p \leq .001$; improving is defined as scoring at least 0.5 points lower and worsening is defined as scoring at least 0.5 points higher on the pregnancy-related anxiety subscale at follow-up compared to baseline.

© 2025 Geusens F. and Skalkidou A.
